# Pyruvate Kinase M2 Upregulation Is Associated With Guillain–Barré Syndrome Risk and Immune Dysregulation: Insights From Mendelian Randomization and the Experimental Autoimmune Neuritis Model

**DOI:** 10.1002/brb3.71632

**Published:** 2026-07-31

**Authors:** Shuping Liu, Huiying Lu, Yuge Kang, Zheman Xiao, Qian Min, Yi Dong

**Affiliations:** ^1^ Department of Neurology Renmin Hospital of Wuhan University Wuhan China; ^2^ Cancer center Renmin Hospital of Wuhan University Wuhan China

**Keywords:** experimental autoimmune neuritis, methylation, PKM2, Th17 cells

## Abstract

**Objective:**

This study aimed to investigate the potential involvement of pyruvate kinase M2 (PKM2) in the pathogenesis of Guillain–Barré syndrome (GBS).

**Methods:**

Mendelian randomization (MR) analysis was used to evaluate the causal association between PKM2 expression and GBS susceptibility, with mediation analysis performed to explore immune cell‐mediated pathways. The association between PKM2 methylation and GBS risk was further examined using GoDMC mQTL data and GRT‐qPCRWAS Data Hub profiling. To validate these findings experimentally, an experimental autoimmune neuritis (EAN) mouse model was established in male C57BL/6 mice (6–8 weeks) by immunization with P0180–199 peptide, with tissues collected at disease initiation (Day 8) and peak stage (Day 16). PKM2 expression dynamics, Th17/Treg (regulatory T cells) balance, and histopathological changes were assessed using, Western blot, immunohistochemistry, immunofluorescence, flow cytometry, and H&E/Luxol fast blue (LFB) staining.

**Results:**

MR analysis revealed that elevated PKM2 expression in the tibial nerve was significantly associated with an increased risk of GBS (OR: 1.75–6.14, *p* < 0.05). In the EAN model, PKM2 expression in the sciatic nerve exhibited a time‐dependent increase (*p* < 0.01), primarily localized to inflammatory infiltrates. Mechanistic studies demonstrated that PKM2 upregulation in CD4+ T cells was associated with promoted Th17 differentiation, as evidenced by the upregulation of IL‐17a and RORγt (*p* < 0.05), concomitant with a reduction in Treg cells (*p* < 0.01). To further elucidate the upstream epigenetic regulation, the association between PKM2 methylation and GBS risk was investigated using GoDMC mQTL data. Among the four selected methylation sites, two‐sample MR analyses indicated that cg24327132 were positively associated with GBS risk (*p* < 0.05). Furthermore, analysis of the GWAS Data Hub revealed that cg24327132 appeared to be preferentially hypomethylated in adaptive immune cells including regulatory T cells, CD4+ T cells, and B cells (Beta ≈ 0–0.05), visually distinct from innate immune populations (Beta ≈ 0.10–0.20), suggesting that perturbation of this hypomethylated state may dysregulate PKM2 expression and contribute to GBS susceptibility.

**Conclusion:**

PKM2 upregulation is associated with GBS risk and immune dysfunction. PKM2 methylation may represent a key regulatory mechanism underlying this association. PKM2 may represent a mechanism target warranting further therapeutic investigation.

## 1 Introduction

Guillain–Barré syndrome (GBS) is an acute autoimmune disorder affecting the peripheral nervous system, marked by the demyelination of peripheral nerves and nerve roots, along with the presence of inflammatory cell infiltration (Shahrizaila et al. 2021). It is the most common cause of flaccid paralysis worldwide. Due to the potential for severe complications such as respiratory failure and autonomic nerve disorders, approximately one‐third of GBS patients require admission to the intensive care unit (Hughes 2020). Intravenous immunoglobulin (IVIG) and plasmapheresis are the only clinically effective treatments proven to date, but both are expensive and have limited effects (Shang et al. 2021). Therefore, further exploration of the pathogenesis of GBS and improvement of patient prognosis are particularly urgent.

As the most common pathological subtype of GBS, the pathogenesis of acute inflammatory demyelinating polyneuropathy (AIDP) is still unclear (Yuki and Hartung 2012). Although scholars continue to explore the immune response cascade involved in the development of AIDP, attempting to find specific antibody markers, there has been no breakthrough progress (Miyaji et al. 2014). Currently, scholars believe that the immune mechanism of this subtype is mainly involved in cellular immunity (van den Berg et al. 2014).

Th17 cells are an important part of adaptive immunity, help the host resist extracellular pathogens, and are related to the pathogenesis of autoimmune‐mediated inflammatory diseases (such as systemic lupus erythematosus and ankylosing spondylitis) (Yasuda et al. 2019). Our previous research showed that the increase in Th17 cell differentiation is an important mechanism in the development of experimental autoimmune neuritis (EAN), a rat model of AIDP (Liu et al. 2019). There are also studies that use the PCR‐RFLP method for genetic typing of the G197A single nucleotide polymorphism in the IL17 gene of 220 GBS patients, observing increased plasma IL‐17A levels in GBS patients (Debnath et al. 2018). These studies add new dimensions and emphasize the importance of the Th17 inflammatory pathway. However, the regulatory signaling pathways that control the differentiation and pathogenicity of Th17 cells are still unclear.

Based on previous research reports, Th17 cells show significant metabolic reprogramming characteristics during differentiation or activation, with their energy metabolism shifting from oxidative phosphorylation to aerobic glycolysis (Papadopoulou and Xanthou 2022). Pyruvate kinase (PK) catalyzes the conversion of phosphoenolpyruvate to pyruvate, which is a key step (Lunt et al. 2015). The M2 type of PK (PKM2), as the final rate‐limiting enzyme of the glycolytic pathway, not only supports cell proliferation by catalyzing glycolysis but also has the ability to regulate gene expression (Kono et al. 2019). It is worth noting that PKM2 is not essential for the metabolic reprogramming and proliferation of Th17 cells, and T cell‐specific PKM2 deficiency inhibits Th17‐mediated inflammatory responses by reducing Th17 cell differentiation. Moreover, the absence of PKM2 in T cells selectively inhibits the differentiation of Th17 cells without affecting the differentiation of Th1, Th2, or iTreg cells in vitro (Angiari et al. 2020). Therefore, PKM2, as a key non‐metabolic regulatory factor, fine‐tunes the differentiation and function of Th17 cells in autoimmune‐mediated inflammation. However, to date, no studies have explored whether PKM2 is involved in the pathogenesis of AIDP/EAN.

This study intends to explore whether PKM2 is involved in the pathogenesis of GBS through a Mendelian randomization (MR) analysis. To validate this hypothesis, EAN model was used to investigate possible mechanisms.

## 2 Materials and Methods

### 2.1 Animals Model

A total of 18 male C57BL/6 mice, aged 6–8 weeks (20 g), were obtained from Beijing Vital River Laboratory Animal Technology Co., Ltd. (license no. SCXK‐Jing‐2016‐0011). All animals were maintained under SPF conditions with 5–6 mice per cage. Environmental parameters were controlled at 22°C ± 2°C with 50% ± 10% relative humidity and a 12‐h light/dark cycle. Standard rodent chow and sterilized water were provided ad libitum. A 1‐week acclimatization period was observed before any experimental procedures. All experimental procedures involving animals were approved by the Animal Research Ethics Committee of Renmin Hospital of Wuhan University (approval number: 202510483). Euthanasia was performed by carbon dioxide (CO_2_) inhalation at a displacement rate of 20% of the chamber volume per minute (5 L/min), in accordance with the AVMA Guidelines for Euthanasia (American Veterinary Medical Association (AVMA) 2020).

EAN was induced via subcutaneous injection into the dorsal region of mice on Day 0 and Day 8. Each injection contained 150 µg of P0 peptide 180–199 (SSKRGRQTPVLYAMLDHSRS; GL Biochem Ltd., Shanghai, China) and 0.5 mg of *Mycobacterium tuberculosis* (strain H37 RA; Difco, Detroit, Michigan, USA) emulsified in a mixture of 25 µL saline and 25 µL Freund's incomplete adjuvant (FIA; Sigma, USA). Additionally, all mice received 400, 300, and 300 ng of pertussis toxin (PTX; Merck, Whitehouse Station, New Jersey, USA) via tail vein injection on Days −1, +1, and +3, respectively.

Mice were randomly assigned to three groups using a random number table: a control group (in which PBS was substituted for P0_180–199_ during EAN induction), an EAN model group euthanized on Day 8 postimmunization (p.i.) (EAN 8d), and an EAN model group euthanized on Day 16 p.i. (EAN 16d), with six mice per group. Clinical signs and scores were assessed and recorded daily for all groups. Upon completion of the experiment, spleen and sciatic nerve tissues were collected for subsequent analysis.

### 2.2 Functional Testing and Gait Assessment

The assessment of disease progression was conducted by two independent investigators. Clinical scoring was performed immediately prior to immunization (designated as Day 0) and subsequently on a daily basis until the endpoint of the experiment. Disease severity was graded according to the following criteria—0: normal; 1: reduced activity and diminished tail tonus; 2: completely flaccid tail; 3: impaired gait; 4: ataxic gait; 5: mild paraparesis; 6: moderate paraparesis; 7: severe paraparesis; with a score of 0.5 assigned for intermediate clinical manifestations.

Gait analysis was performed using the DigiGait imaging system (eMouse specifics, Inc., Framingham, Massachusetts, USA); mice were recorded while running on a motorized transparent treadmill. A standardized running protocol was implemented, requiring mice to maintain locomotion for 36 s at a constant belt speed of 15 cm/s. Animals unable to sustain running throughout this duration were considered to have failed the task. All recorded sessions were analyzed with DigiGait 8VR software to extract quantitative gait parameters. Testing was conducted at 48‐h intervals, with particular focus on hind limb gait width as a key metric of locomotor function.

### 2.3 Flow Cytometry Analysis

The spleens and sciatic nerve were removed under aseptic conditions at different phases. Following enzymatic digestion with collagenase, DNase, or hyaluronidase to degrade the extracellular matrix, the tissue was mechanically dissociated using a syringe plunger through a 40‐µm cell strainer to generate a single‐cell suspension. After eliminating red blood cells, cell viability and total cell counts were determined using a hemocytometer with trypan blue exclusion. Only samples yielding a minimum of 1 × 10^8^ viable cells were proceeded for subsequent staining. Fc blocking solution was used to prevent nonspecific antibody binding. Cells were resuspended at a concentration of 1 × 10^7^ cells/mL, and 100 µL of cell suspension (1 × 10^6^ cells) was used per staining reaction. For immunophenotyping, CD4+ T cells were labeled with anti‐CD4 (Cat# 11‐0041‐81, Invitrogen, California, USA) and anti‐CD25 antibodies (Cat# 17‐0251‐81, Invitrogen, California, USA); Th17 cells with anti‐CD4 and anti‐IL‐17 (Cat# 17‐7177‐81, Invitrogen, California, USA); and regulatory T cells (Treg) with anti‐CD4, anti‐CD25, and anti‐FoxP3 antibodies (Cat# 12‐5773‐80, Invitrogen, California, USA). The proportions of Th17 and Treg cells were quantified on the flow cytometer (CytoFLEX, Beckman Coulter, Inc., USA). A minimum of 1 × 10^5^ events within the gated population were acquired for each sample to ensure analytical reliability. The gating strategies for the identification of Th17 and Treg cells are presented in Figure S1.

### 2.4 Quantitative reverse transcription polymerase chain reaction (RT‐qPCR))

Total RNA was extracted from splenic CD4+ T cells and sciatic nerve tissues using TRIzol reagent (Ambion, California, USA) according to the manufacturer's instructions. RNA concentration and purity were assessed by NanoDrop spectrophotometry. Complementary DNA was synthesized from 500 ng total RNA using the Reverse Transcriptase kit (Vazyme Biotech, Nanjing, China). RT‐qPCRwas performed on a ViiA 7 RT‐PCR System (Applied Biosystems, California, USA) using SYBR Green Master Mix under the following conditions: 95°C for 10 min, followed by 40 cycles of 95°C for 15 s and 60°C for 1 min. Relative gene expression was calculated using the 2−ΔΔCT method with GAPDH as the internal reference gene. Primer sequences are listed in Table 1.

**TABLE 1:** Primers used for RT‐qPCR.

Gene	Forward (5'→3')	Reverse (5'→3')
**PKM2**	GCCGCCTGGACATTGACTC	CCATGAGAGAAATTCAGCCGAG
**IL‐17a**	TTTAACTCCCTTGGCGCAAAA	CTTTCCCTCCGCATTGACAC
**IL‐6**	TAGTCCTTCCTACCCCAATTTCC	TTGGTCCTTAGCCACTCCTTC
**TGF‐β**	CTCCCGTGGCTTCTAGTGC	GCCTTAGTTTGGACAGGATCTG
**IL‐23**	CCAGCAGATTCAGAATCCTGAG	GAACTCAGTGCTGGCACTAAG
**RORα**	CCCTTCACACACAGCCTCAC	TGGGCATAAGTCCTTGCACA
**RORγt**	TGTGCTGCCTACCTCTGACC	TGACAGTTCCACCTCCTCGT
**HIF‐1α**	AGCTTCTGTTATGAGGCTCACC	TGACTTGATGTTCATCGTCCTC
**HK2**	TGATCGCCTGCTTATTCACGG	AACCGCCTAGAAATCTCCAGA
**LDHA**	CATTGTCAAGTACAGTCCACACT	TTCCAATTACTCGGTTTTTGGGA
**GLUT1**	CAGTTCGGCTATAACACTGGTG	CCCTAGCAGTTGGCTATAAGCT
**GAPDH**	AGGTCGGTGTGAACGGATTTG	GGGGGCTAAGCAGTTGGTG

### 2.5 Western Blot

Total protein was extracted from sciatic nerve tissues using RIPA lysis buffer (containing protease and phosphatase inhibitors). Protein concentration was determined using a BCA protein assay kit (Cat# AS1086, ASPEN, Wuhan, China). Equal amounts of protein (20–30 µg) were separated by 10% SDS‐PAGE and then transferred onto PVDF membranes. After blocking with 5% nonfat milk in TBST for 1 h at room temperature, the membranes were incubated with anti‐PKM2 antibody (1:1000; Cat# ABC438, Sigma–Aldrich, St. Louis, Missouri, USA) overnight at 4°C. The next step is incubation with HRP‐conjugated secondary antibody (Cat# A6154, Sigma–Aldrich, St. Louis, Missouri, USA) for 2 h at room temperature. The membranes were then stripped and re‐probed with β‐actin.

### 2.6 Immunohistochemistry and Immunofluorescence

Paraffin‐embedded sciatic nerve sections (4 µm) were deparaffinized and rehydrated using standard procedures. Antigen retrieval was performed by heating in sodium citrate buffer (10 mM, pH 6.0) at 95°C for 20 min, followed by inactivation of endogenous peroxidase with 3% hydrogen peroxide for 10 min at room temperature. Sections were blocked with 5% BSA for 30 min and incubated overnight at 4°C with anti‐PKM2 primary antibody (1:200; Cat# ABC438, Sigma–Aldrich, St. Louis, Missouri, USA). For immunohistochemistry, sections were incubated with biotin‐conjugated secondary antibody (1:200; Sanying Biotechnology, Wuhan, China) for 1 h at room temperature, followed by DAB chromogen development and hematoxylin counterstaining. For immunofluorescence, sections were incubated with FITC‐labeled secondary antibody (1:200; Sanying Biotechnology, Wuhan, China) for 1 h at room temperature, followed by DAPI counterstaining (Sigma–Aldrich, USA). Slides were mounted and examined under an inverted fluorescence microscope.

### 2.7 H&E Staining and Luxol Fast Blue (LFB) Staining

Sciatic nerve tissues were fixed in 4% paraformaldehyde, dehydrated, and embedded in paraffin. Sections of 4 µm thickness were prepared for histological analysis. For H&E staining, sections were deparaffinized, stained with Harris hematoxylin (Sigma, USA) for 5–10 min, differentiated in 1% acid–alcohol, blued under running water, and counterstained with eosin for 1–2 min. Inflammatory cell infiltration was quantified by counting the number of infiltrating cells per square millimeter (n/mm^2^) in three randomly selected fields per section at 400× magnification using ImageJ software. For LFB staining, deparaffinized sections were immersed in 0.1% LFB solution (95% ethanol with 0.05% acetic acid) at 56°C–60°C overnight (16 h), differentiated sequentially in 0.05% lithium carbonate solution and 70% ethanol until the myelinated areas appeared bright blue and the background was colorless, and counterstained with cresyl violet. The severity of demyelination was scored using a semiquantitative grading system: 0, normal; 1, less than 25% demyelinated fibers; 2, 25%–50% demyelinated fibers; 3, 50%–75% demyelinated fibers; 4, more than 75% demyelinated fibers. The demyelinating scores were measured independently by two observers in a blinded manner.

### 2.8 MR Data Sources

We employed MR to investigate the causal relationship between PKM2 in the human genome and GBS. The eQTL dataset was downloaded from the GTEx Portal database (https://www.gtexportal.org/home/) as the exposure data. We acquired the finngen_R12_G6_GUILBAR dataset from the FINNGEN database as the outcome data for GBS, consisting of 551 cases and 491,583 controls (https://storage.googleapis.com/finngen‐public‐data‐r12/summary_stats). GWAS summary statistics for 731 immune phenotypes, including B cells, dendritic cells, mature T cells, and monocytes, were retrieved from the GWAS Catalog (accession numbers GCST90001391‐GCST90002121) (Orrù et al. 2020). The mQTL summary statistics were obtained from the GoDMC mQTL meta‐analysis (http://mqtldb.godmc.org.uk/downloads). Both the exposure and outcome datasets were sourced from European populations, ensuring uniformity and coherence of the data. Since the MR analysis in this study utilized publicly available GWAS summary data, no additional ethical approval or informed consent was required.

### 2.9 Selection of Instrumental Variables (IVs)

The selection of IVs was based on specific criteria. Using the “TwoSampleMR” package, we evaluated the eQTL dataset for each gene and identified SNPs strongly associated with gene expression levels (*p* < 5 × 10^−^^8^). If no genome‐wide significant SNPs were available as IVs, a more lenient threshold (*p* < 1 × 10^−^^5^) was applied. And sensitivity analyses for these weak instruments become crucial in further study. The *F*‐statistic quantified the joint ability of genetic instruments to explain variation in the exposure, and we set an *F*‐statistic threshold of 10 for the selected variants to avoid bias from weak instruments. The clumping technique (*r* ^2^ = 0.001 and clumping distance = 10,000 kb) was used in the present work to assess the extent of LD among the contained SNPs (Hemani et al. 2018). For the 731 immune phenotypes used as exposures, SNPs were selected as IVs if the *p*‐value was less than 1 × 10^−5^ (Carter et al. 2021).

### 2.10 MR Analysis and Mediation MR Analysis

Following the identification of IVs, we applied two‐sample MR analysis to assess the causal relationship between exposure (e.g., PKM2 and immune cells) and outcome (GBS) using “TwoSampleMR” package, with a focus on results derived from the inverse variance weighted (IVW) method as the primary analytical method, which offered the most precise evaluation in the absence of horizontal pleiotropy (Sekula et al. 2016). Additionally, four other MR methods, namely, MR–Egger, weighted median, simple mode, and weighted mode, were employed as supplementary approaches to IVW. Unless stated otherwise, the MR results given in this publication were based on the results of IVW. The findings on causal relationships were ultimately expressed as odds ratios (OR) together with their corresponding 95% confidence interval (CI). The significance criterion was established at a value of 0.05. Therefore, to make the results between exposure and outcome more stable and reliable, testing for horizontal pleiotropy was necessary. The MR–Egger intercept test was employed to evaluate horizontal pleiotropy, with a significance threshold set at *p* < 0.05. Deviation of the MR–Egger intercept from zero indicated the existence of horizontal pleiotropy. Additionally, due to variations in the selected population and experimental conditions, there might be heterogeneity among samples. Therefore, Cochran's *Q* test was used to detect heterogeneity (*p* > 0.05), and a leave‐one‐out analysis was performed to assess the consistency of the pleiotropy test results. We also discarded those unclear and repeated SNPs, and the alleles were incorporated into the human genome reference sequence (build 37).

Furthermore, we employed a two‐step MR to perform mediation analysis in R software (4.3.1), aiming to evaluate the potential role of immune cell phenotypes as intermediaries in the causal pathway linking the PKM2 to GBS risk. First, the causal relationships between eQTLs and immune cell traits were analyzed, followed by the associations between immune cells and GBS. The total effect (*β*) quantified the overall PKM2‐GBS relationship, while *β*1 represented the effect of the PKM2 on immune cells, and *β*2 represented the effect of immune cells on GBS. The mediating (indirect) effect was estimated as *β*1 × *β*2. The proportion mediated was calculated using the formula as follows: (mediation effect / total effect) × 100%, with a 95% CI provided (Carter et al. 2021).

### 2.11 Statistical Analysis

Statistical analyses were performed using GraphPad Prism 8. Continuous variables are expressed as mean ± standard deviation (SD). Between‐group comparisons were conducted using unpaired *t*‐tests or one‐way ANOVA, with repeated‐measures ANOVA applied where appropriate. A *p*‐value < 0.05 was considered statistically significant.

## 3 Results

### 3.1 MR Analysis Using Nerve eQTL Data Confirmed That High Expression of PKM2 in the Sciatic Nerve Was Significantly Associated With the Risk of GBS

**FIGURE 1:**
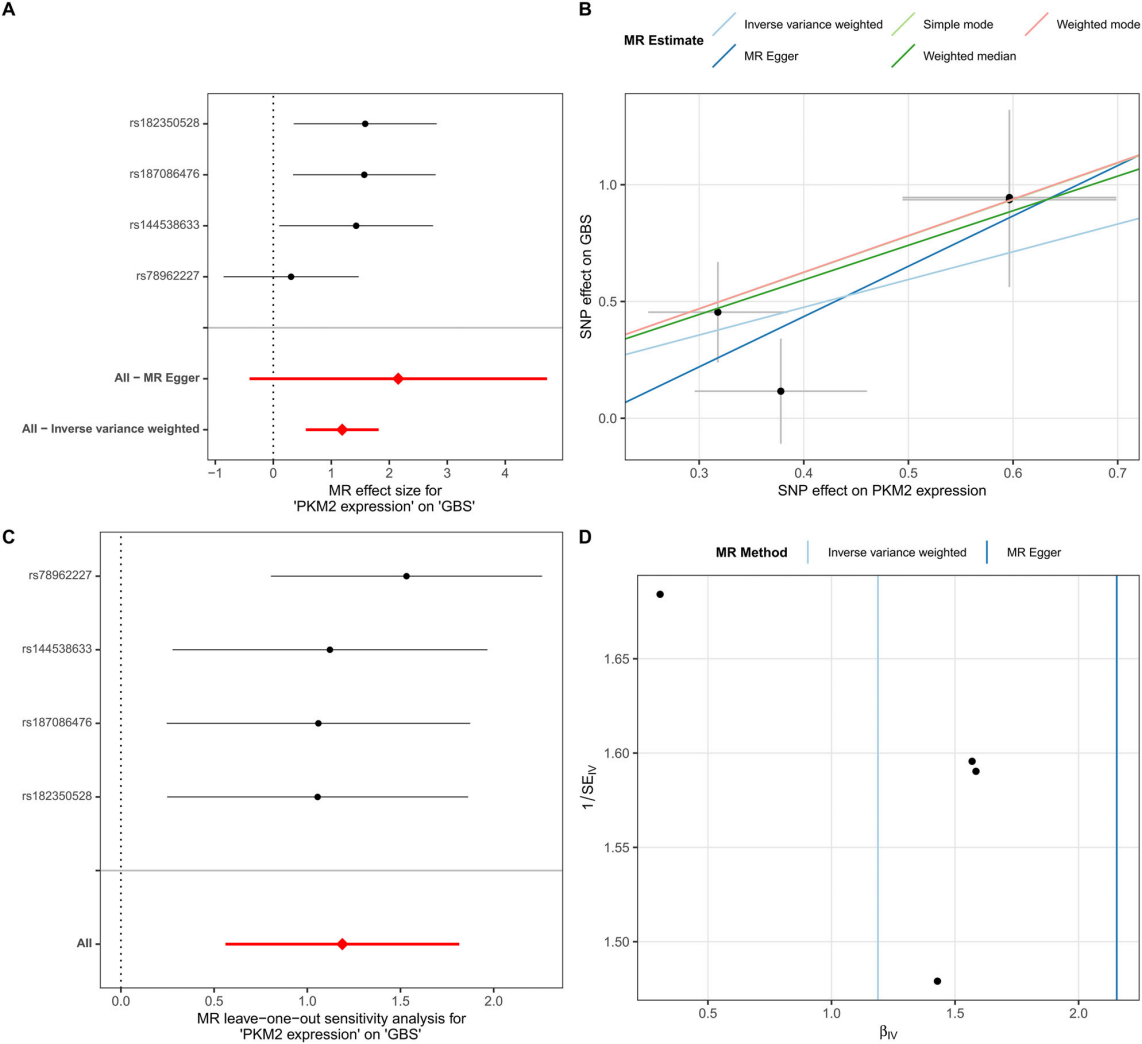
MR analyses for PKM2 on GBS. (A) Forest plot: The individual SNPs show varying effects and confidence intervals. The overall MR estimates (red lines) suggest that there might be a positive association from the IVW method. (B) Scatter plots: Individual inverse variance (IV) associations with PKM2 displayed individual IV associations with GBS in black dots. The 95% CI of odd ratio for each IV is shown by vertical and horizontal lines. The slope of the lines represents the estimated causal effect of the MR methods. (C) Leave‐one‐out analysis: Based on the tight clustering of the red line around a similar value across the different SNP removals, the overall MR estimate appears relatively robust. (D) Funnel plot.

To investigate the causal role of PKM2 in GBS, we performed MR analysis using PKM2 expression in the tibial nerve as the exposure and GBS as the outcome. MR analysis identified a significant positive causal association between genetically predicted PKM2 expression and GBS risk. Across multiple MR methods, effect estimates were broadly consistent: IVW (OR = 3.281, 95% CI = 1.753–6.140, *p* < 0.001), weighted median (OR = 4.398, 95% CI = 1.999–9.677), and simple mode (OR = 4.761, 95% CI = 1.408–16.105), supporting a robust positive causal effect. Sensitivity analyses confirmed the reliability of these findings. The MR–Egger intercept test showed no evidence of directional pleiotropy (*β* = −0.426, SE = 0.557, *p* = 0.524). Cochran's *Q* test indicated no significant heterogeneity across instruments, in either the IVW model (*Q* = 3.10, df = 3, *p* = 0.377) or the MR–Egger model (*Q* = 2.40, df = 2, *p* = 0.302). Together, these results support a causal role for PKM2 upregulation in increasing GBS susceptibility (Figure 1).

### 3.2 Clinical and Pathological Features of EAN

**FIGURE 2:**
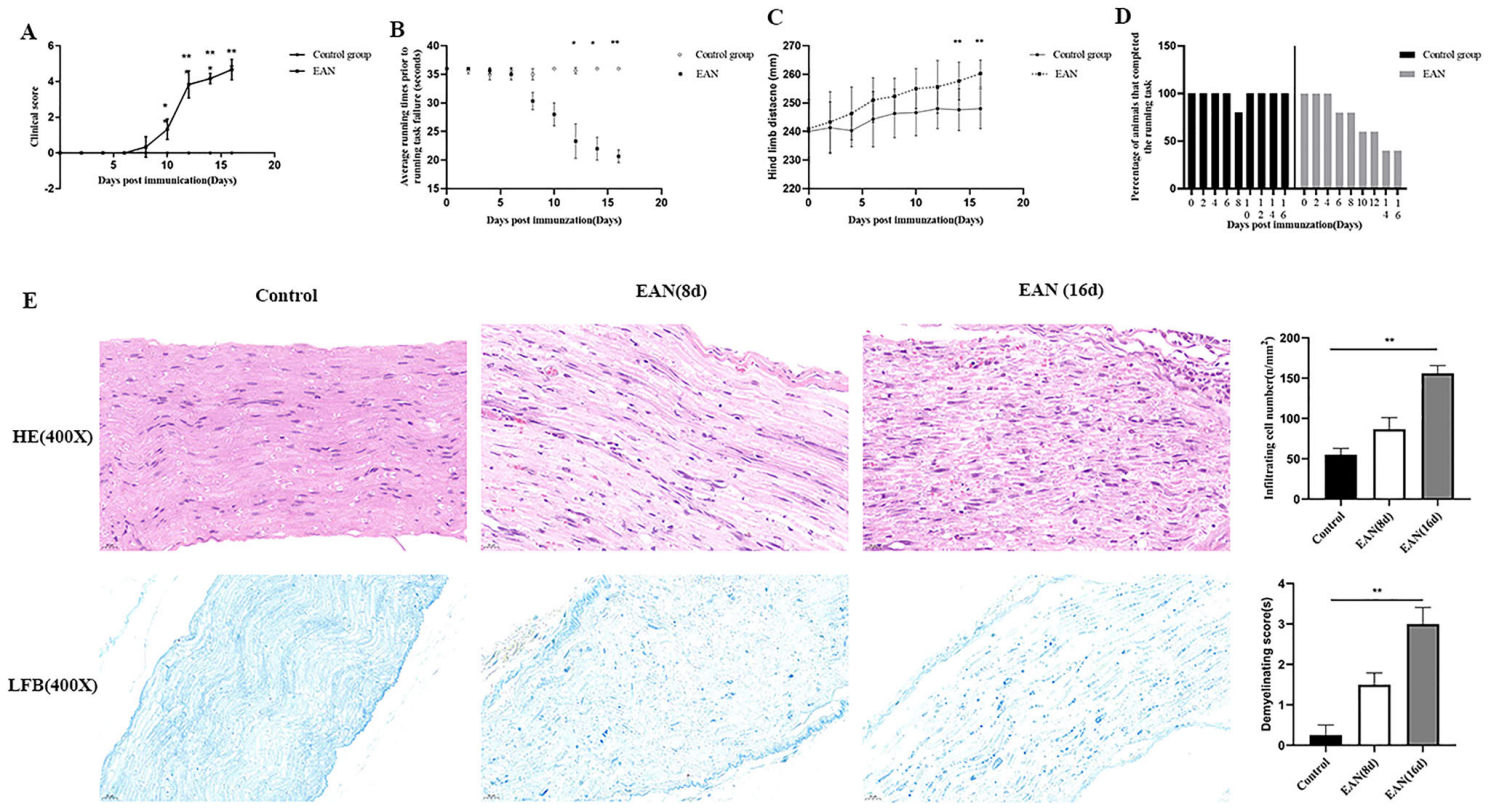
(A) Clinical scores for EAN versus healthy controls. (B) Average running times prior to running task failure. (C) Plot of hind limb gait width versus time. (D) Percentage of mice that completed the running task over the course of disease. (E) Sciatic nerve under H&E and LFB staining (400×) (*p < 0.05; **p < 0.01; ***p < 0.001).

C57BL/6 mice with EAN exhibited significant clinical deficits compared with control mice, as evidenced by progressively elevated clinical scores (*p* < 0.01) (Figure 2A). Motor function assessed by treadmill running revealed a significant reduction in time to failure in EAN mice compared to controls (*p* < 0.05) (Figure 2B), with the first failures observed as early as Day 6 p.i. (Figure 2C). No control mice consecutively failed the running task and there was a 100% completion rate at the end stage of the assessment period. Given that motor deficits were detectable prior to clinical score changes, Day 8 p.i. was selected as the early disease time point for subsequent analyses. Gait analysis using the DigiGait system further demonstrated a significant progressive increase in hindlimb gait width in EAN mice (*p* < 0.01), which was absent in control animals (Figure 2D), providing an additional objective measure of motor impairment.

Histopathological examination of sciatic nerves confirmed successful EAN model establishment. H&E staining revealed pronounced structural disorganization, wavy distortion of nerve fibers, and mononuclear cell infiltration in EAN mice compared to controls. LFB staining demonstrated marked demyelination in EAN mice compared to controls (Figure 2E). Collectively, these functional and histopathological findings are consistent with the characteristic features of EAN and confirm reliable disease induction.

### 3.3 The Expression of PKM2 in Sciatic Nerve of EAN Showed a Time‐Dependent Increase During the Disease Process

**FIGURE 3:**
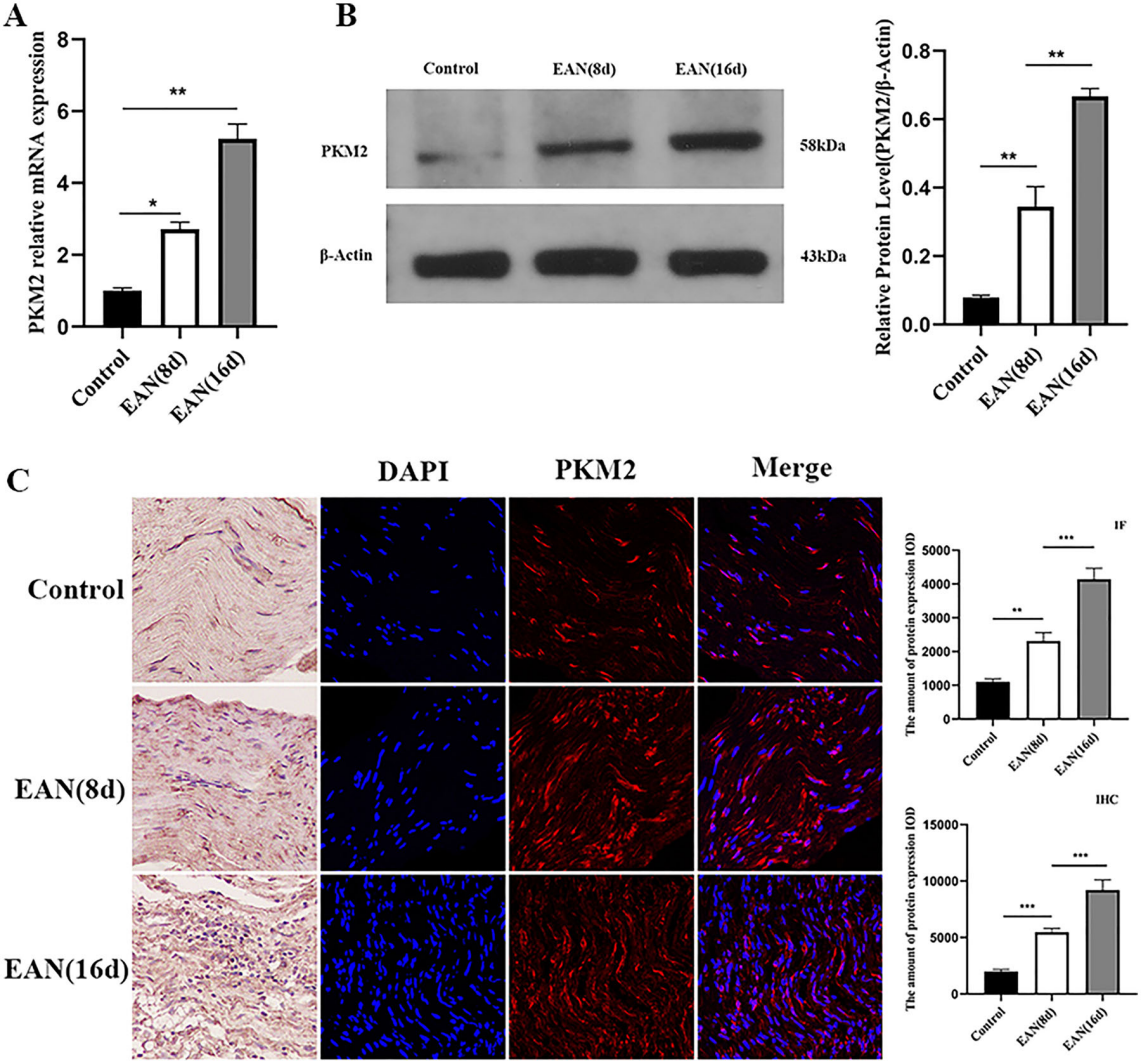
(A) RT‐qqPCR shows the changes of PKM2 mRNA expression in sciatic nerve during the progression of EAN mice. (B) WB detection of changes in the protein expression levels of PKM2 in sciatic nerve. (C) PKM2 expression in the sciatic nerve was analyzed by immunohistochemistry and immunofluorescence (red); DAPI was used as a nuclear marker (blue). Scale bar represents 50 µm(*p < 0.05; **p < 0.01; ***p < 0.001).

We then analyzed the dynamic expression of PKM2 in EAN disease progression. We found that the expression of PKM2 mRNA in sciatic nerve gradually increased at the early stage of the disease (Day 8) and the peak stage of symptoms (Day 16) (*p* < 0.01) (Figure 3A). WB detection of changes in the protein expression levels of PKM2 in sciatic nerve showed the same trend (Figure 3B). Immunohistochemistry further confirmed that the level of PKM2 protein was elevated in the sciatic nerve of EAN mice (Figure 3C). Additionally, immunofluorescence staining indicated that the expression of PKM2 was almost confined to the areas of inflammatory cell infiltration, and was almost absent in the sciatic nerve sections of control mice.

### 3.4 The Potential Role of Immune Cells Between PKM2 and GBS/EAN

To investigate whether the causal effect of PKM2 on GBS is mediated through specific immune cell populations, we performed a two‐step mediation MR analysis integrating PKM2 eQTLs with 731 immune cell traits. Initially, standard two‐sample MR identified 41 immune cell traits causally associated with GBS, the majority of which belonged to Tregs, myeloid cells, and B cells (Table S1). Among these, HLA DR++ monocyte %leukocyte (OR = 0.661, 95% CI = 0.502–0.871, *p* = 0.003) and transitional B cell absolute count (transitional AC) (OR = 0.706, 95% CI = 0.544–0.917, *p* = 0.009) exhibited protective effects against GBS (Table S2). However, subsequent two‐step mediation analysis revealed that neither of these two immune traits exerted a statistically significant mediating effect. Specifically, the total causal effect (*β*) of PKM2 on GBS was 0.780. The indirect effect mediated through HLA DR++ monocyte %leukocyte was −0.101 (*p* = 0.121), while the mediation proportion through transitional AC was estimated at 7.21% (*p* = 0.243) (Table 2). These results indicate that while these immune cell subsets show a nominal suggestive trend, they do not conclusively mediate the causal pathway between PKM2 and GBS in the current framework, warranting further validation in larger cohorts.

**TABLE 2:** The mediation effect of immune cells on the causal effect of PKM2 on GBS.

Mediator	Total effect *β*	Direct effect *β*	Indirect effect *β*12	*Z*	*p*	Mediation proportion
HLA DR++ monocyte %leukocyte	0.780	0.881	−0.101	−1.551	0.121	—
Transitional AC	0.780	0.724	0.056	1.167	0.243	7.21%

**FIGURE 4:**
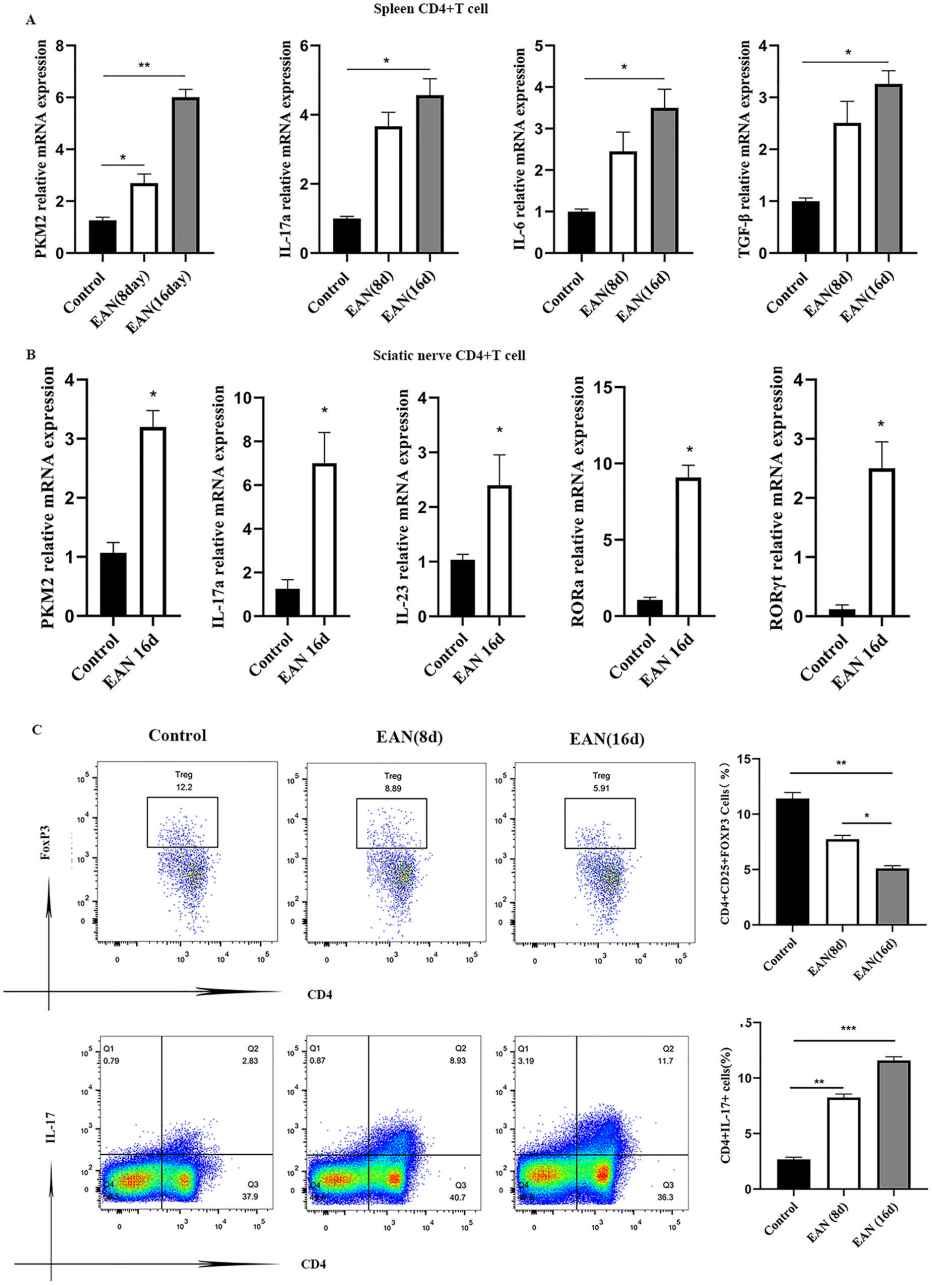
(A) RT‐qPCR shows the changes of PKM2, IL‐17a, IL‐6, TGF‐β mRNA expression in spleen CD4+ T cell during the progression of EAN mice. (B) RT‐qPCR shows the changes of PKM2 and Th17‐related genes (IL‐17a, IL‐23, RORa, RORγt) in CD4+ T cells of the sciatic nerve during the progression of EAN mice. (C) Dynamic changes of Th17 cells and Tregs cells in the spleen during the disease process(*p < 0.05; **p < 0.01; ***p < 0.001).

Then, we further explored the immune microenvironment of EAN. We found that the expression of PKM2 mRNA in CD4+ T cells of the spleen significantly increased with the progression of the disease (*p* < 0.01), accompanied by the increase in the expression of IL‐17a, IL‐6, and TGF‐β mRNA (Figure 4A). The transcriptional levels of PKM2 and Th17‐related genes (Il17a, Il23, Rora, Rorc) in CD4+ T cells of the sciatic nerve were significantly upregulated (*p* < 0.05) (Figure 4B). The analysis of immune cells in the spleen showed that the proportion of Th17 cells increased while that of Tregs decreased (*p* < 0.01) (Figure 4C), suggesting that the high expression of PKM2 participates in the pathogenesis of GBS/EAN by promoting Th17 differentiation.

### 3.5 Methylated Regulation on PKM2 Expression Influencing GBS

**FIGURE 5:**
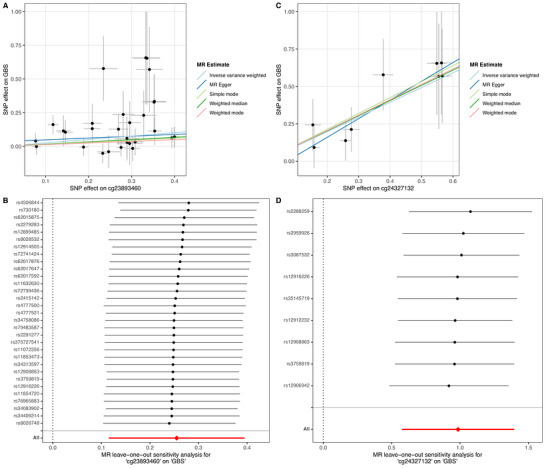
MR analyses for PKM2 methylation sites on GBS. (A) Scatter plots for cg23893460. (B) Leave‐one‐out analysis for cg23893460. (C) Scatter plots for cg24327132. (D) Leave‐one‐out analysis for cg24327132.

To further elucidate the upstream regulatory mechanisms of PKM2 in GBS, we investigated the causal relationship between PKM2 methylation and GBS risk using GoDMC mQTL data. Among the 41 candidate methylation sites, four loci, including cg09485853, cg11028091, cg23893460, and cg24327132, were selected for two‐sample MR analysis. Positive associations with GBS risk were identified for cg23893460 and cg24327132 by IVW (*p* < 0.05); however, the robustness of these associations differed markedly across methods (Figure 5 and Table S3).

For cg23893460, the primary IVW method yielded a nominally significant positive association (OR = 1.29, *p* < 0.001); however, this was not consistently replicated across other MR methods (all *p* > 0.05), limiting confidence in this finding. Sensitivity analyses showed no evidence of horizontal pleiotropy (*p* = 0.905) or outlier‐driven bias (MR‐PRESSO, *p* = 0.546), suggesting that the inconsistency across methods reflects limited statistical power rather than methodological bias.

In contrast, cg24327132 demonstrated robust and consistent evidence of a positive causal association with GBS risk across all MR methods: IVW (OR = 2.68, *p* < 0.001), MR–Egger (OR = 3.49, *p* = 0.037), weighted median (OR = 2.76, *p* < 0.001), and simple mode (OR = 2.90, *p* = 0.027). The absence of horizontal pleiotropy (*p* = 0.568) and outlier influence (MR‐PRESSO, *p* = 0.973) further strengthens the credibility of this association, supporting a reliable causal role for cg24327132 methylation in GBS susceptibility.

For the remaining two loci, cg09485853 and cg11028091, no statistically significant associations were identified by any MR method (IVW: *p* = 0.848 and *p* = 0.237, respectively), and the overall evidence did not support a causal relationship with GBS risk.

**FIGURE 6:**
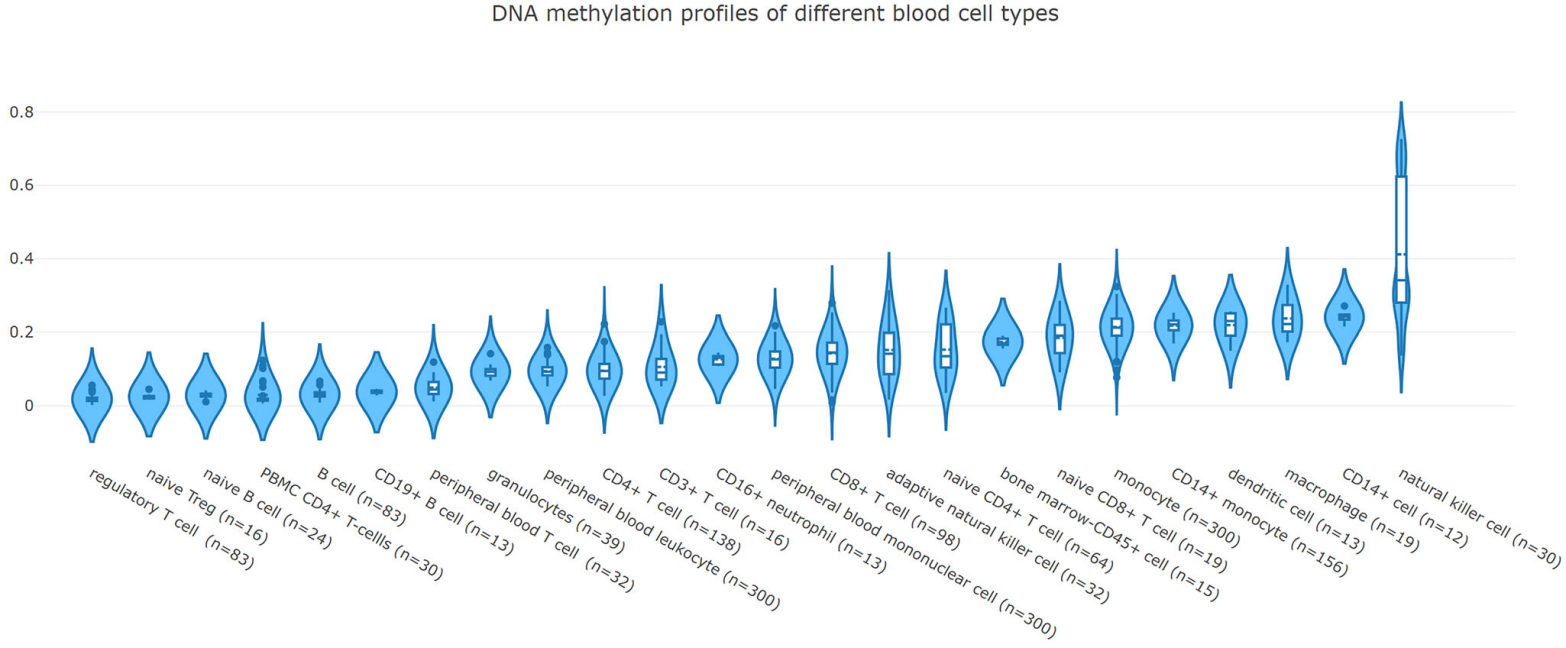
DNA methylation for cg24327132 of different blood cell types.

To investigate the cell type‐specific methylation pattern of cg24327132, we analyzed its methylation profile across 25 blood cell types using the EWAS Data Hub. All samples used to assess methylation levels of tissues were normal or adjacent normal. As shown in Figure 6, cg24327132 exhibited significantly lower methylation levels in adaptive immune cell subsets (Beta ≈ 0–0.05), including Tregs, naïve Treg cells, naïve B cells, CD4+ T cells, and B cells, compared with innate immune populations such as monocytes, dendritic cells, and macrophages (Beta ≈ 0.10–0.20). These findings indicate that cg24327132 is maintained in a hypomethylated state in GBS‐relevant immune cells, suggesting that genetic variants perturbing this hypomethylated state may upregulate PKM2 expression and contribute to GBS susceptibility.

## 4 Discussion

While PKM2 has been extensively studied in cancer and CNS autoimmunity (Wiese and Hitosugi 2018; Yang et al. 2024), its role in peripheral nervous system autoimmunity has not previously been characterized. To elucidate the role of elevated PKM2 expression in the pathogenesis of GBS, this study employed MR analysis to establish a causal association between elevated PKM2 expression in tibial nerve tissue and increased GBS susceptibility. By leveraging genetic variants as IVs, MR minimizes confounding and reverse causation inherent to conventional observational studies, thereby providing stronger causal inference (Burgess et al. 2023).

The temporal upregulation of PKM2 in sciatic nerves of EAN model further corroborates its active role in AIDP/EAN pathogenesis. Rather than serving as a static biomarker, PKM2 expression increased progressively from disease initiation to peak stage. Immunofluorescence staining revealed that PKM2 immunoreactivity was almost exclusively confined to areas of inflammatory cell infiltration in EAN sciatic nerves, and was virtually absent in control specimens. These findings suggest that PKM2 induction is not merely an epiphenomenon of neuroinflammation, but may actively drive disease progression through immune cell‐intrinsic mechanisms.

To systematically investigate the potential mediating role of immune cells in the gene‐disease association, we employed mediation MR analysis. Our results identified HLA DR++ monocytes and transitional B cells as potential mediators of the PKM2–GBS relationship, both exhibiting significant protective effects against GBS. HLA DR++ monocytes, characterized by high antigen‐presenting capacity, play critical roles in maintaining immune homeostasis and controlling autoimmunity (Liu et al. 2023). Transitional B cells suppress autoreactive CD4+ T cell proliferation, inhibit Th17 differentiation, and promote FoxP3+ Treg generation (Flores‐Borja et al. 2013), functions directly relevant to the Th17/Treg imbalance observed in our EAN model. PKM2 upregulation may therefore partially promote GBS susceptibility by suppressing these regulatory immune populations. Although neither mediating effect reached statistical significance, our findings contribute novel insights into the potential immunomodulatory role of immune cells in gene–disease associations, prompting our further investigation into the immune microenvironment in EAN.

From the perspective of immune cell subset differentiation, the dynamic balance between Th17 cells and Tregs is primarily regulated by key cytokines, including IL‐6 and TGF‐β (Bettelli et al. 2006). Our data revealed a significant upregulation of PKM2 mRNA in splenic CD4+ T cells during disease progression, concomitant with elevated expression of IL‐17a, IL‐6, and TGF‐β mRNA. Furthermore, CD4+ T cells isolated from the sciatic nerves of peak‐stage EAN mice exhibited higher levels of PKM2 mRNA and Th17‐associated genes (IL‐17a, IL‐23, Rora, and Rorγt) compared to those from control mice. These findings suggest that PKM2 may serve as a regulator of the Th17/Treg axis in EAN. We acknowledge that PKM2 has also been reported to coordinate metabolic reprogramming and enhance effector functions in CD8+ T cells (Mortazavi Farsani et al. 2025). Interestingly, contrary to the initial hypothesis that PKM2 upregulation would drive downstream glycolytic reprogramming in CD4+ T cells, we did not observe any significant increase in other genes related to glycolysis in CD4+ T cells during the disease process of EAN mice (Figure S2). This suggests that PKM2‐driven Th17 differentiation in EAN may operate through a non‐metabolic mechanism independent of glycolytic reprogramming. Supporting this interpretation, Damasceno et al. (2020) demonstrated that PKM2 promotes Th17 differentiation by translocating into the nucleus and interacting with STAT3 to enhance its activation, a process entirely independent of glycolytic gene regulation. Alternatively, in the context of the complex in vivo environment of EAN, where CD4+ T cells represent a heterogeneous mixture of naïve, effector, and memory subsets, the metabolic signature of the minority Th17 population may be insufficient to drive statistically detectable changes in bulk glycolytic gene expression, even following magnetic‐bead enrichment for CD4+ T cells. Future studies employing single‐cell RNA sequencing or Th17‐specific isolation would provide greater resolution of the metabolic reprogramming occurring within pathogenic T cell subsets in EAN.

To elucidate the upstream regulation of PKM2 in GBS, we investigated the relationship between PKM2 methylation and disease risk using GoDMC mQTL data. The identification of cg24327132 as a methylation locus robustly associated with GBS risk across all MR methods provides novel epigenetic evidence for PKM2 dysregulation in this disease. DNA methylation predominantly occurs at CpG dinucleotides, influencing transcription by altering chromatin structure and accessibility, and dysregulated methylation patterns have been identified across multiple autoimmune diseases, with hypomethylated CpG sites often associated with enhanced gene expression in pro‐inflammatory immune cells (Zhang et al. 2025). The positive association between methylation at cg24327132 and GBS risk may reflect a site‐specific paradoxical regulatory mechanism, whereby hypermethylation at these discrete loci disrupts the binding of transcriptional repressors, thereby enhancing rather than silencing PKM2 expression.

Analysis of cg24327132 methylation profiles across 25 blood cell types in the EWAS Data Hub revealed that this locus is maintained in a constitutively hypomethylated state in GBS‐relevant adaptive immune cells, including Tregs, CD4+ T cells, and B cells. This lineage‐specific hypomethylation suggests that cg24327132 is subject to cell type‐specific epigenetic programming that maintains PKM2 in a transcriptionally permissive state in adaptive immune cells, rendering PKM2 expression in these populations particularly sensitive to upstream genetic perturbations. Unlike genetic changes, epigenetic aberrations are reversible, which provides a direction for disease treatments by pharmaceutically targeting dysregulated epigenetic regulation (Younesian et al. 2024). This reversibility renders the PKM2 methylation loci identified in this study attractive as candidates for epigenetic intervention in GBS.

Several limitations of this study warrant acknowledgment. First, male C57BL/6 mice were exclusively used, as this strain and sex combination is the only one demonstrated to be susceptible to P0 180–199–induced EAN (Zou et al. 2000; Li et al. 2024). Since GBS affects individuals of both sexes, the generalizability of our findings to female subjects remains to be established. Future studies employing female models with alternative induction strategies would be valuable to address this limitation. Second, although MR analysis identified cg24327132 as a methylation site positively associated with GBS risk, the precise transcriptional regulatory elements governed by this locus, and the mechanisms by which genetic variants alter PKM2 expression in disease‐relevant immune cells, remain to be elucidated. DNA methylation sequencing of clinical GBS samples would represent the ideal approach to validate these epigenetic findings and clarify the functional relevance of cg24327132 in human disease. Third, while our data implicate PKM2 non‐metabolic pathway as the likely mechanistic basis for Th17 promotion, direct functional validation through T cell‐specific PKM2 deletion or TEPP‐46 treatment in the EAN model would further strengthen this conclusion.

## 5 Conclusion

PKM2 upregulation is associated with GBS risk and immune dysfunction. PKM2 methylation at specific CpG loci may represent an upstream regulatory mechanism. These findings nominate PKM2 as a candidate target for future functional and therapeutic investigation, pending direct validation through loss‐of‐function and rescue experiments.

## Author Contributions

**Shuping Liu**: **Shuping Liu**: conceptualization, methodology, data curation, investigation, software, validation, formal analysis, supervision, funding acquisition, visualization, project administration, resources, Writing – original draft, Writing – review and editing. **Huiying Lu**: investigation, validation, formal analysis, writing – original draft, methodology, software, project administration. **Yuge Kang**: methodology, software, investigation, validation, formal analysis, writing – original draft, project administration. **Zheman Xiao**: methodology, software, investigation, writing – review and editing. **Qian Min**: conceptualization, methodology, software, data curation, investigation, validation, formal analysis, supervision, visualization, project administration, writing – review and editing. **Yi Dong**: conceptualization, methodology, software, data curation, investigation, validation, formal analysis, supervision, visualization, project administration, writing – review and editing.

## Funding

This study was supported by the National Natural Science Foundation of China (grant no. 82201488) and Natural Science Foundation of Hubei Province (grant no. 2025AFD513).

## Ethics Statement

Animal studies were approved by The Animal Research Ethics Committee of Remin Hospital of Wuhan University (a copy of the ethics approval form was shown in attached file).

## Conflicts of Interest

The authors declare no conflicts of interest.

## Supporting information




**Supplementary Figure**: brb371632‐sup‐0001‐Figure1.pdf

**Supplementary Figure**: brb371632‐sup‐0002‐Figure2.tif

**Supplementary Table**: brb371632‐sup‐0003‐TablesS1‐S3.xlsx

## Data Availability

The data used to support the findings of this study are available from the corresponding author upon request.
