# Supplementary figures and images for: Pyruvate Kinase M2 Upregulation Is Associated With Guillain–Barré Syndrome Risk and Immune Dysregulation: Insights From Mendelian Randomization and the Experimental Autoimmune Neuritis Model
Source: Brain Behav. 2026 Jul 31;16(8):e71632. doi: 10.1002/brb3.71632 (PMC13425601; doi:10.1002/brb3.71632)

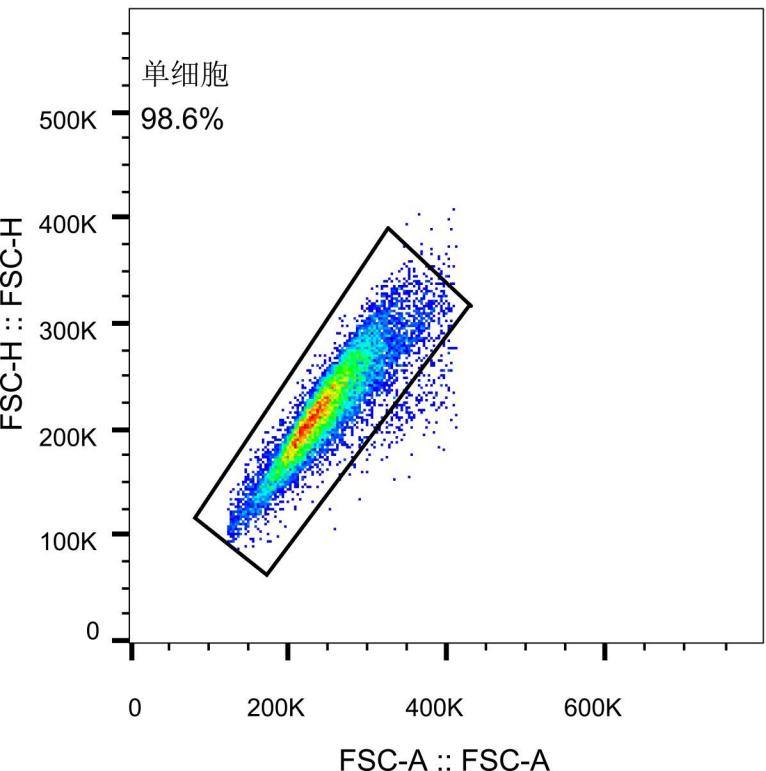

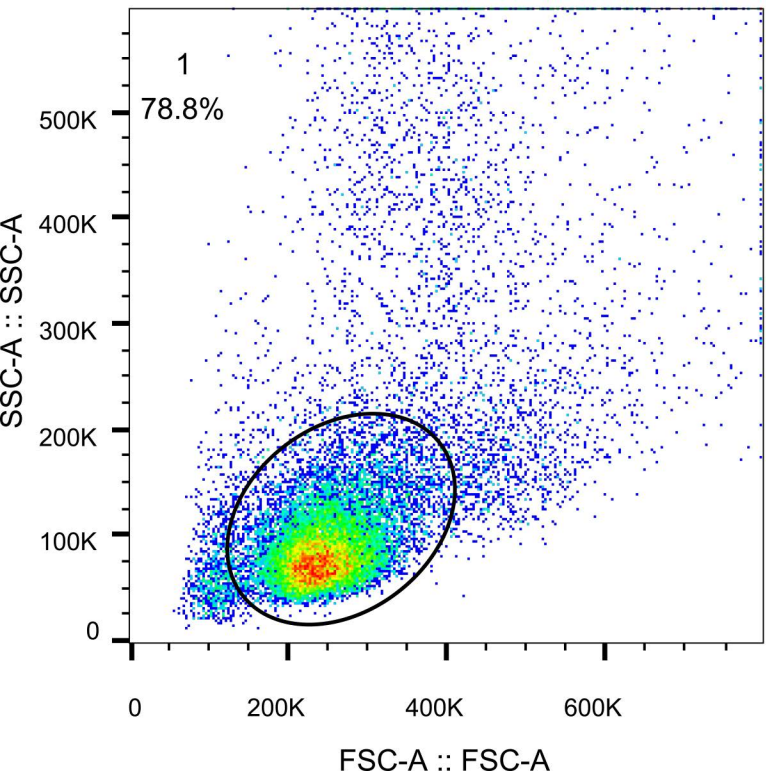

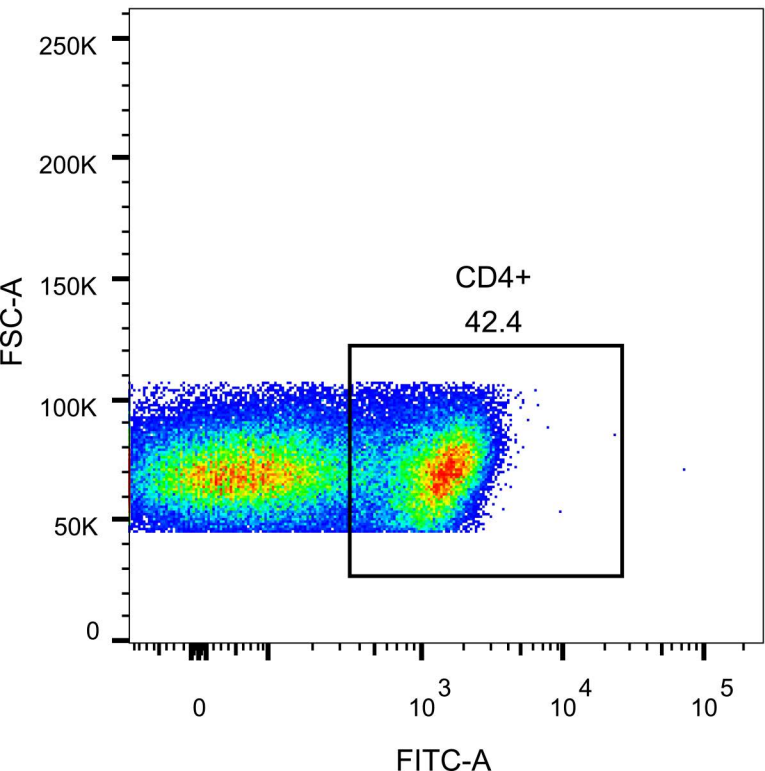

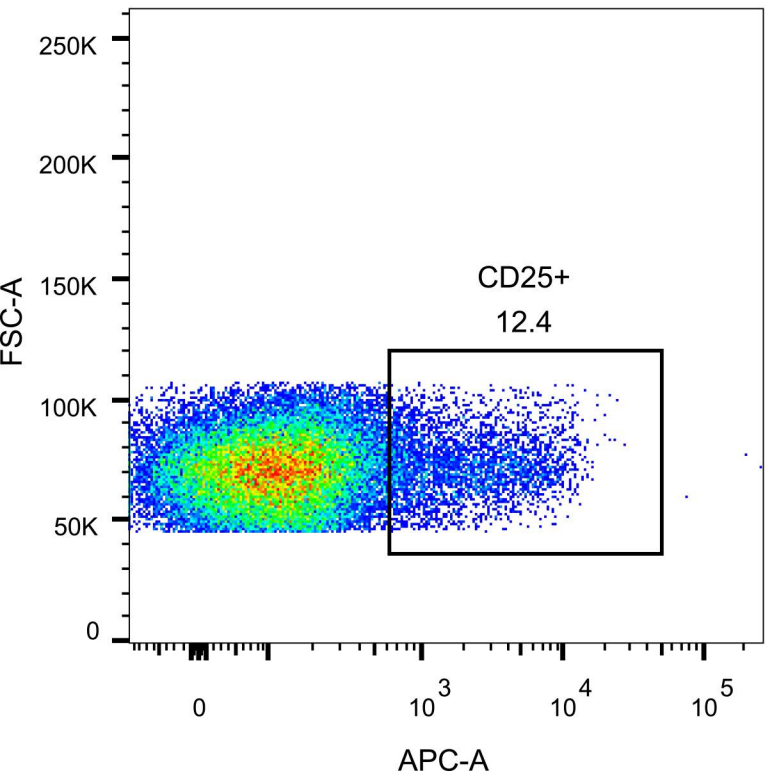

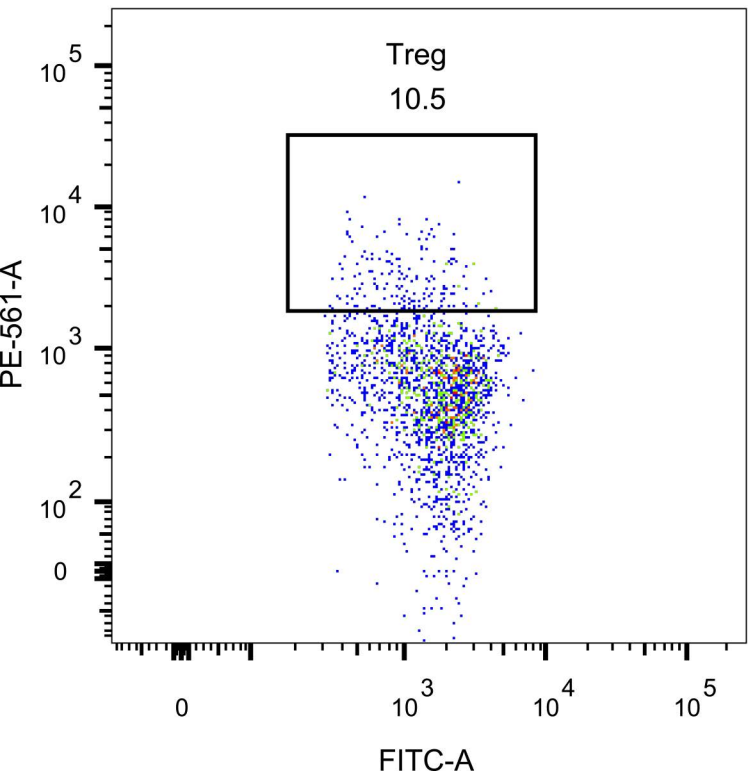

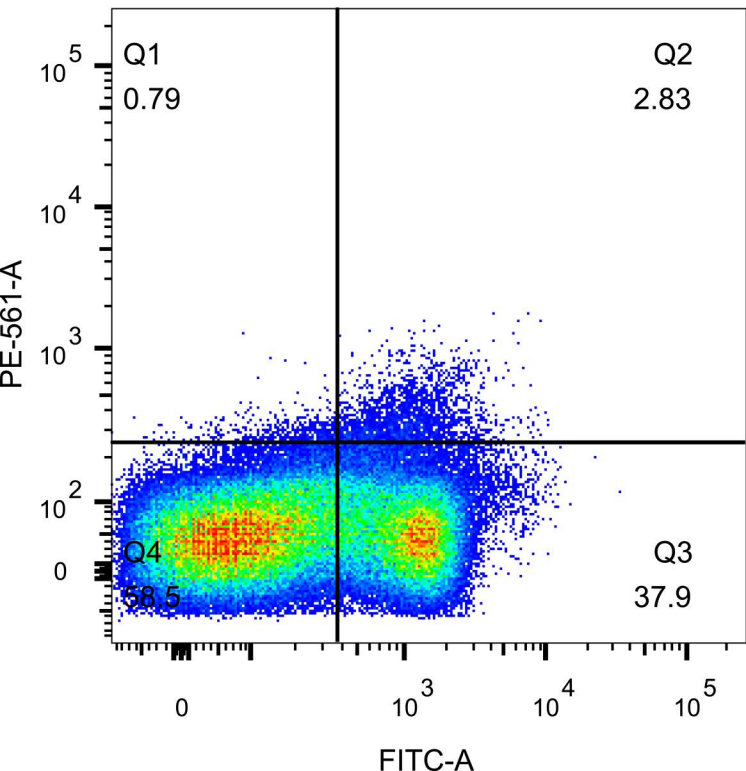

Supplement: Supplementary file 1 — Supplementary Figure: brb371632‐sup‐0001‐Figure1.pdf [file BRB3-16-e71632-s003.pdf]

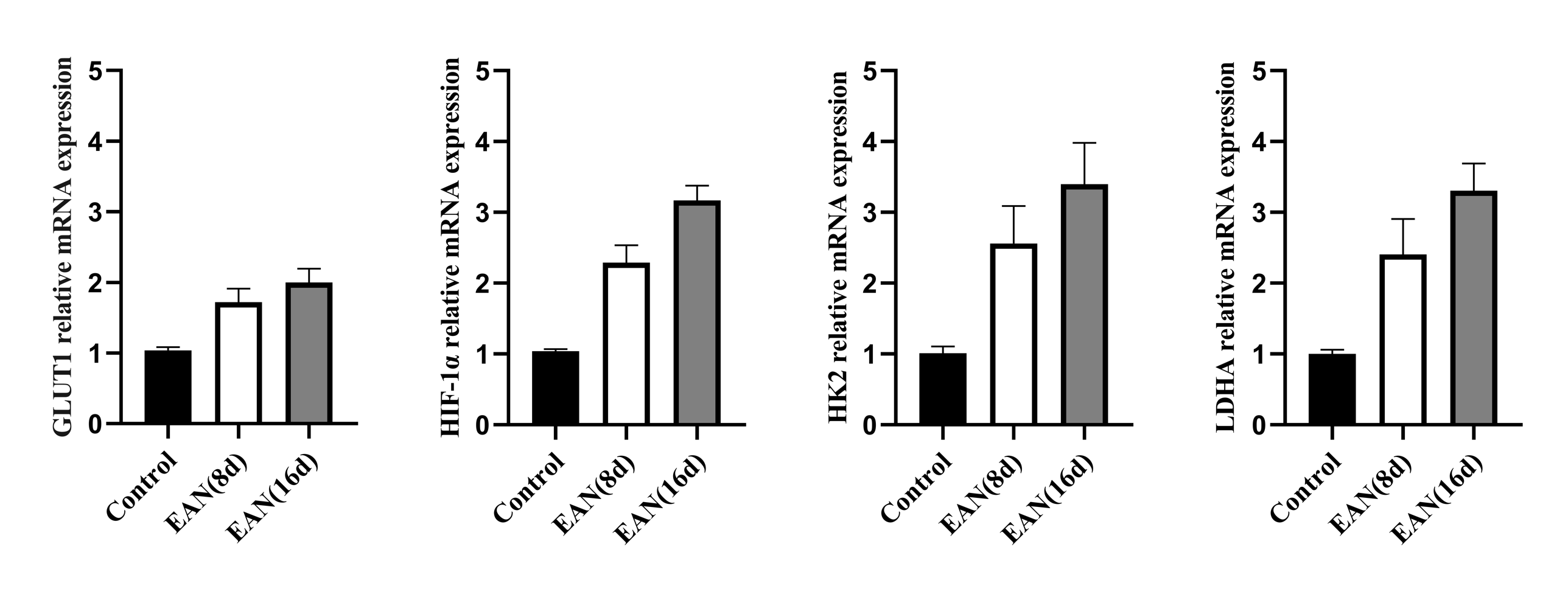

Supplement: Supplementary file 2 — Supplementary Figure: brb371632‐sup‐0002‐Figure2.tif [file BRB3-16-e71632-s002.tif]
